# Preparation of Porous Hydroxyethyl Cellulose Materials to Utilize Lactic Acid with Vacuum-Assisted Process

**DOI:** 10.3390/molecules28093702

**Published:** 2023-04-25

**Authors:** Haeun Lee, Do Chun Nam Kung, Sang Wook Kang

**Affiliations:** 1 Department of Electrical Engineering, Sangmyung University, Seoul 03016, Republic of Korea; 2 Department of Chemistry and Energy Engineering, Sangmyung University, Seoul 03016, Republic of Korea

**Keywords:** hydroxyethyl cellulose, lactic acid, separator, porosity

## Abstract

For the first time, we succeeded in manufacturing a 2-hydroxyethyl cellulose (HEC)-based composite membrane with improved thermal stability, for use as a battery separator, coating a HEC polymer solution to a polypropylene (PP) support and using a vacuum-assisted process. A HEC polymer solution was prepared by utilizing HEC and lactic acid (LA) as a plasticizer. A vacuum-assisted process was used to move ethanol, which a mobile phase to permeate a plasticized region in the HEC polymer side for pore formation. The pores formed with uniform nano sizes, and areas in which some large pores formed were observed. The thermal stability of the composites was measured using TGA. The thermal decomposition temperatures were measured at about 250 °C for the neat HEC, about 210 °C for the HEC/LA film, and about 335 °C for the HEC/LA/PP membrane before the process. After the vacuum-assisted process, the first and second thermal decomposition were observed at about 360 °C and 450 °C, respectively. The HEC/LA/PP membrane after the process showed greater thermal stability than before the process. This means that the adhesion between the HEC polymer and the PP support was created through the rearrangement of the HEC chain, as LA escaped after the process, and it was seen indirectly that the mechanical strength was enhanced. In particular, the surface of the membrane was observed by SEM to investigate whether the HEC penetrated into the PP to block its pores, and whether the HEC region collapsed. Furthermore, the interaction of the HEC chain with the additives and the rearrangement of the HEC was confirmed using FT-IR. As a result, we demonstrated that the mechanical strength and thermal stability of the manufactured HEC/LA/PP membrane were enhanced.

## 1. Introduction

Recently, the battery industry has become very important through the rapid development of electric vehicles and portable electronic devices [1,2]. In particular, lithium-ion batteries (LIBs) are among the most widely used power sources for energy-storage systems due to their long lifespan, low self-discharge, and high energy density [3]. However, LIBs are less thermally stable than other battery systems. Therefore, many fire accidents caused by electric vehicles and mobile phones have recently occurred [4]. The major reason for this is presumed to be the separator, which porous the polymer membrane is used to prevent direct contact between the cathode and the anode [5]. The separators used in LIBs are polyolefin materials, such as polyethylene and polypropylene. These materials offer many advantages, such as chemical stability and uniform pore distribution, but they also have low electrolyte absorption and ionic conductivity, and their low thermal stability causes internal short circuits and safety issues at high temperatures [6,7,8,9]. Therefore, it is important to develop separators with good thermal stability and mechanical properties [10].

There are two ways to solve the problems described above. The first involves manufacturing a composite separator by coating an organic/inorganic material on the surface of a polyolefin separator [11]. In the case of a separator coated with Al_2_O_3_, it is more hydrophilic than PE-separator surfaces, the wettability is improved by polar liquid electrolytes. Improved wettability affects both liquid-electrolyte absorption and ionic conductivity. In addition, the interfacial resistance of the battery is reduced, and the battery has the ability to retain more electrolytes in the separator than when PE separators are used. Therefore, ion shortage and leakage can be prevented [12]. However, these methods do not solve all problems. Another option is to develop a new type of membrane. The polyimide (PI)/SiO_2_ composite separator not only combines the excellent thermal stability of polymers, but also exhibits the high wettability and excellent mechanical properties of silica [13]. The silica layer imparts hydrophilicity to the outside of the composite separator and greatly improves the wettability of liquid-electrolyte diffusion. However, this is an expensive method due to the electrospinning and the various other processes involved. Therefore, studies on the manufacturing of separators have been actively conducted, with a particular focus on cellulose series, which are cheap and eco-friendly biodegradable natural polymers with high wettability, and good thermal stability [14,15,16,17].

On the other hand, porous polymer membranes that uses a battery separator are produced in various ways, and various manufacturing methods are being developed. In particular, the phase-inversion method is mainly used in the field of membrane manufacturing. Non-solvent-induced phase separation (NIPS) and thermally induced phase separation (TIPS) are generated through interactions between polymers and solvents. In NIPS, an asymmetrically shaped surface is created, while in TIPS, a porous and symmetrical structure is created. The use of NIPS is effective in reducing the ion-migration resistance inside the battery due to changes in the electrolyte concentration inside the separator. The TIPS method is relatively simple and easy to manufacture, since a separation membrane is formed in the process of mixing and cooling the polymer solution. In addition, it has the advantages of good reproducibility and the low occurrence of defects. However, both methods have the disadvantage that pores are randomly formed and the curve is very large, meaning that the movement distance of the lithium ions becomes long [18]. This affects the charging speed of the battery. In order to increase the charging speed of the battery, there is a method for reducing the moving distance by making the pore shape of the separator straight. The track-etch method [19] previously used for manufacturing straight-shaped pores, is a very expensive process using radiation.

Therefore, in our laboratory, a new approach was found, in which a linear separator is manufactured cellulose acetate (CA)/specific additives composite membrane with a hydraulic method and specific additives. In this process, metal salts are used as additives. However, it has a disadvantage, in that the cost is high. Additionally, many salts, such as Zn and Ni, are utilized, and the required energy is relatively high [20,21]. Furthermore, there is an additional disadvantage in that it is difficult to apply uniform pressure, and the associated costs are relatively high. In addition, CA with a molecular weight of 30,000 g/mol was used, and its mechanical strength is confirmed to be weak. To solve these problems, a new cellulose-based material should be developed and it was necessary to introduce new methods, and eco-friendly additives and processes are required.

Therefore, in this study, the aim was to form pores in a separator using 2-hydroxyethyl cellulose (HEC), a cellulose-based material, and lactic acid (LA), an eco-friendly plasticizer. In addition, a vacuum-assisted process was used to support for pore formation using instead of the hydraulic method to reduce costs. HEC with a molecular weight of 90,000 g/mol was used to increase the mechanical strength, which is a disadvantage of CA, 2-hydroxyethyl cellulose (HEC) with a molecular weight of 90,000 g/mol was used. Due to its strong viscosity, HEC is a good candidate for ultra-thin coating due to its strong viscosity [22]. Lactic acid (LA), a plasticizing agent, causes a hydration effect through its strong interaction with water molecules used as a solvent [23]. Therefore, LA was dispersed between the HEC chains to form hydrated parts of various sizes, and it was intended to be converted into pores through the vacuum method. It was confirmed through SEM images that the LA was well dispersed, and uniform pores are formed. In addition, lactic acid is an eco-friendly material produced through fermentation [24,25], and has the advantage of reducing process costs, and is environmentally friendly. Thus, the aim of this study was to analyze the characteristics of porous membranes manufactured using the vacuum-assisted process, which has more advantages than the hydraulic process, and to confirm that it offers improved physicochemical properties.

## 2. Results and Discussion

### 2.1. Ethanol-Flux Data of the HEC/LA/PP Membrane

Table 1 shows the flux data of the ethanol for the mobile phase, during which pores formed in the membrane. These values were determined by the formation of pores in the HEC/LA/PP membrane by the ethanol, the size and number of pores, and the tortuosity inside the membrane. The higher the ethanol-flux value, the faster the ethanol flow. This means that the pore size was large and the internal tortuosity was small. Four reproducible experiments were conducted to obtain the average value. It seems that pores formed in the HEC/LA film and connected to the pores of the PP support. The reason for the formation of pores in the film was that solvation occurred around the lactic acid used as the additive and surrounded the solvent molecules, creating a flexible part. If the flexible phase was inserted and processed using the pressure difference, the channels were connected, since less energy was required to move the flexible phase to the open pores of the PP support than to the blocked parts. In the manufactured separator, the HEC/LA and PP were not separated, even without the use of an adhesive. During the vacuum-assisted process, as the pores were connected, a small amount of HEC/LA permeated into the pores of the PP, the contact-surface area was widened, and bonds appeared to form through the physical interaction between the PP and the HEC/LA. Since HEC/LA is hydrophilic and PP is hydrophobic, it was expected that the film might fall off when the method was applied. However, for the same reasons as those described above, the membrane shape was successfully maintained.

### 2.2. Scanning-Electron Microscope

**Figure 1:**
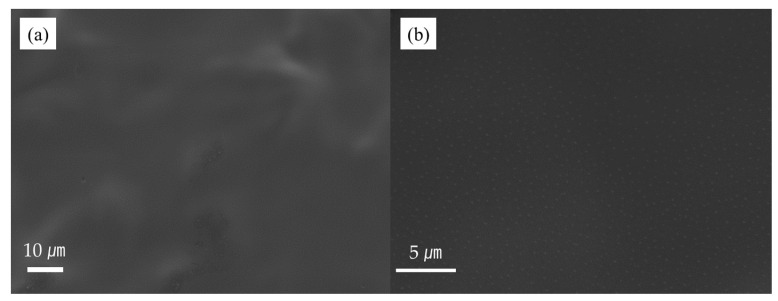
(**a**) HEC side of HEC/LA/PP membrane before process (×1200), and (**b**) enlargement of (**a**) (×4000).

Scanning-electron microscopy (SEM) was used to identify the pores on the film side and the PP side of the HEC/LA/PP composite. As shown in Figure 1a, the entire surface of the film was coated smoothly before the process. At this point, pores had not formed, and the surface was coated smoothly overall. Figure 1b is an enlarged image of Figure 1a. It was confirmed that the numerous round dots on the film surface were evenly spread over the LA plasticized portion.

**Figure 2:**
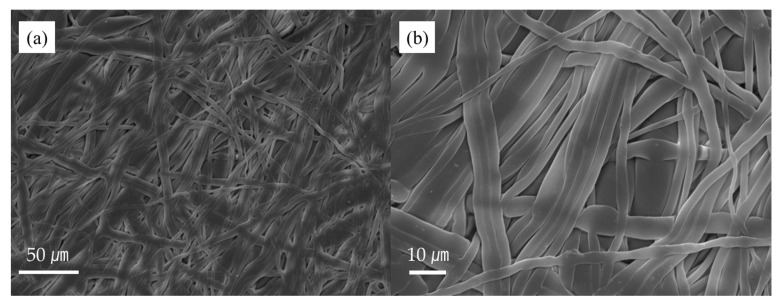
(**a**) PP side of HEC/LA/PP membrane before process (×400), and (**b**) enlargement of (**a**) (×1200).

Figure 2a,b are the PP surface before the application of the method. Since the PP was pressed while it was coated, it was slightly flattened. When coated, the HEC/LA slightly penetrated the PP, but the pores did not collapse. If the pores had collapsed, it could not have been used as a separator, since there would have been no space for the lithium ions to move.

**Figure 3:**
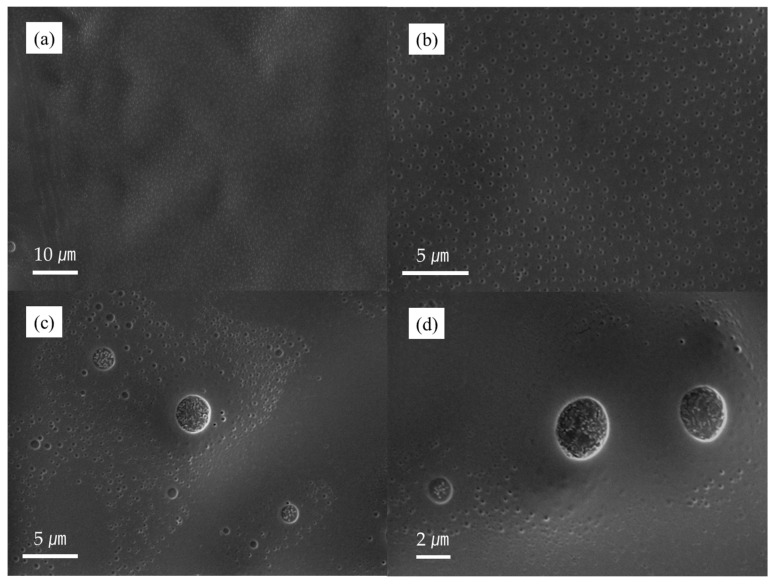
(**a**) HEC side of HEC/LA/PP membrane after process (×1500), (**b**) enlargement of (**a**) (×4500), (**c**) other side of HEC/LA/PP membrane after process (×3700), and (**d**) enlargement of other side (×5500).

Figure 3a–d are images of the HEC/LA/PP membrane’s surface after the vacuum-assisted method was applied. On the surface of the HEC/LA/PP membrane after process, small pores were uniformly formed, as shown in Figure 3a,b. These were distinctly different from those on the surface of the membrane in Figure 1a,b. Furthermore, as shown in Figure 3c,d, large pores partially formed, and the HEC region did not collapse and maintained its morphology, despite the pore formation. Examining the data in Table 1 and the presence or absence of pores in the surface of the membrane, it was confirmed that the pores of the membrane and the pores of the PP were connected, allowing the ethanol to pass through. Furthermore, it was demonstrated that the LA was dispersed well in the HEC, as shown in Figure 1a,b.

**Figure 4:**
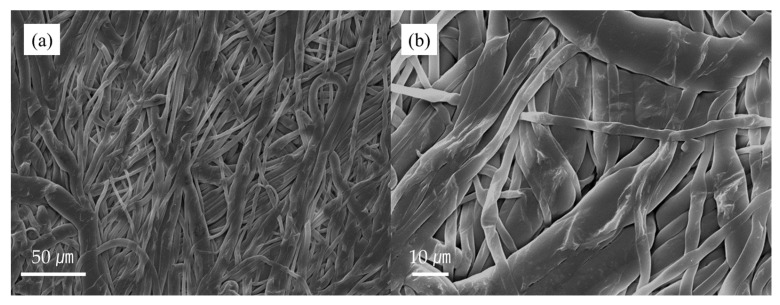
(**a**) PP side of HEC/LA/PP membrane after process (×430), and (**b**) enlargement of (**a**) (×1200).

By examining Figure 4a,b a picture of the PP side after applying the vacuum-assisted process, it can be confirmed that pores were still observable in the PP. After the vacuum-assisted-method, the maintenance of the film indicated that the HEC/LA polymer permeated into the pores of the PP and did not block them.

### 2.3. TGA

**Figure 5:**
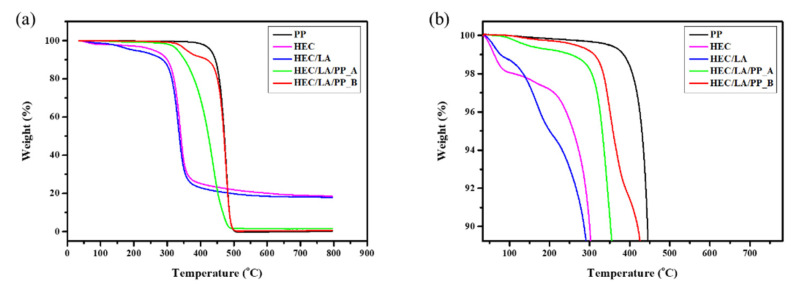
(**a**) TGA data of PP, HEC, and HEC/LA, and (**b**) enlargement of (**a**). HEC/LA/PP_A = before vacuum-assisted process, and HEC/LA/PP_B = after vacuum-assisted process.

The thermal stability of the HEC/LA/PP was investigated through a TGA analysis of the PP, HEC, and HEC/LA (Figure 5a). In Figure 5b, the decomposition temperature of the HEC itself was about 100 °C in the first section and 250 °C for the second. The decomposition temperature of the HEC/LA was about 110 °C in the first section, about 160 °C in the second, and about 210 °C in the third. Since the first decomposition temperature of HEC/LA had a similar decomposition temperature to the pure HEC in the first section, it was thought that the remaining oligomers or solvents decomposed first and, in the second step, it was caused by LA with a low melting point. In the third, it can be seen that the decomposition temperature of the HEC was similar to the secondary decomposition temperature of the HEC/LA and HEC. Comparing the two graphs of the HEC and HEC/LA, it was confirmed that the thermal stability was lower than when the LA was added. When the LA was added to the polymer chains, the polymer was plasticized as the distance between the polymer chains. This induces the decrease of interaction force between the polymer chains. At this point, when exposed to high temperatures, the polymer chains moved more freely, since the LA-added polymers had more free volume than the pure HEC. As a result, the HEC polymer became decomposed more quickly than the pure HEC.

HEC/LA/PP_A decomposition took place at about 114 °C in the first section and at about 335 °C in the second, and in HEC/LA/PP_B, the first and second decompositions were observed at about 360 °C and 450 °C, respectively. The first thermal decomposition of the HEC/LA/PP_A was that of the HEC/LA region. This was attributed to the weight loss due to the evaporation of the remaining solvent or LA in the HEC chain. The second thermal decomposition of the HEC/LA/PP_A featured the decomposition of the HEC and PP. It was confirmed that the HEC and PP were thermally decomposed simultaneously. This seemed to promote the thermal decomposition of the PP support of the HEC, which had a lower thermal decomposition temperature than the PP. As the molten HEC penetrated into the pores of the PP support, it transferred heat directly into the PP. This made the chain of the PP flexible and movable. Therefore, in the HEC/LA/PP_A, the HEC and PP were thermally decomposed together.

The HEC/LA/PP_B thermally decomposed at 360 °C, which was 25 °C higher than the HEC/LA/PP_A. This means that the HEC penetrated into the PP support and adhered to the PP chains. In the first decomposition section, the weight reduced due to the thermal decomposition of the HEC. Subsequently, as the secondary decomposition occurred, similarly to the decomposition temperature of the PP, it can be considered that the PP, as the support of HEC/LA/PP_B, was thermally degraded. Comparing the situations before and after the process, the thermal stability increased after the process. Furthermore, it seems that the LA escaped after the process and that the thermal stability recovered through the recombination of the polymer chains. In other words, as the LA escaped from the HEC polymers during the process, the HEC penetrated further into the PP support and the chains were rearranged, physically attaching to the PP. It was considered that the HEC/LA/PP_B improved the thermal stability. In addition, it was seen indirectly that the mechanical strength was enhanced.

Therefore, the manufacturing of an HEC-based composite membrane for application in battery separators was successful. The LA plasticized the HEC by interfering with the interaction between the HEC chains, escaping through this process, and the HEC rearranged. These findings are confirmed in the following section, on the FT-IR.

### 2.4. Fourier-Transform Infrared Spectroscopy

**Figure 6:**
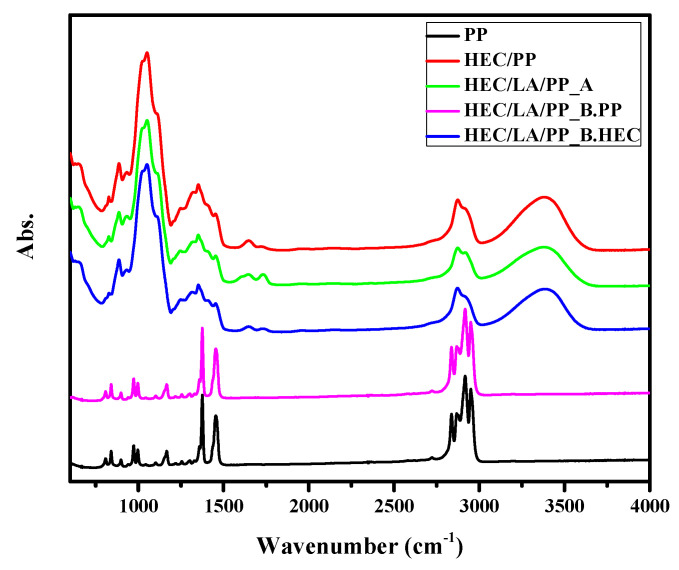
Fourier-transform infrared (FT-IR) spectra of the composites.

**Figure 7:**
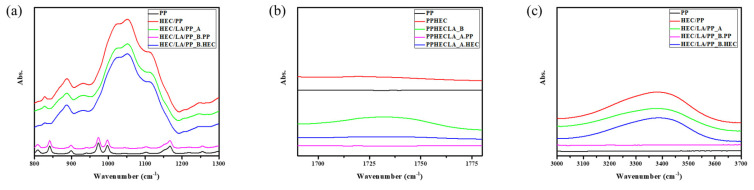
FT-IR spectra: (**a**) ether group, (**b**) carbonyl group, and (**c**) hydroxy group.

To analyze the rearrangement of the HEC and the interaction force between the HEC chain and the LA was observed using FT-IR spectroscopy. (Figure 6) The hydroxy group and the ether group, which were functional groups of the HEC, were investigated (Figure 7a,c) Furthermore, in Figure 7b, since the carbonyl group was found only on the HEC/LA/PP_B graph and was not found on the HEC/LA/PP_A graph, it was considered that the LA was almost entirely escaped during the vacuum-assisted process.

#### 2.4.1. Hydroxy Group

**Figure 8:**
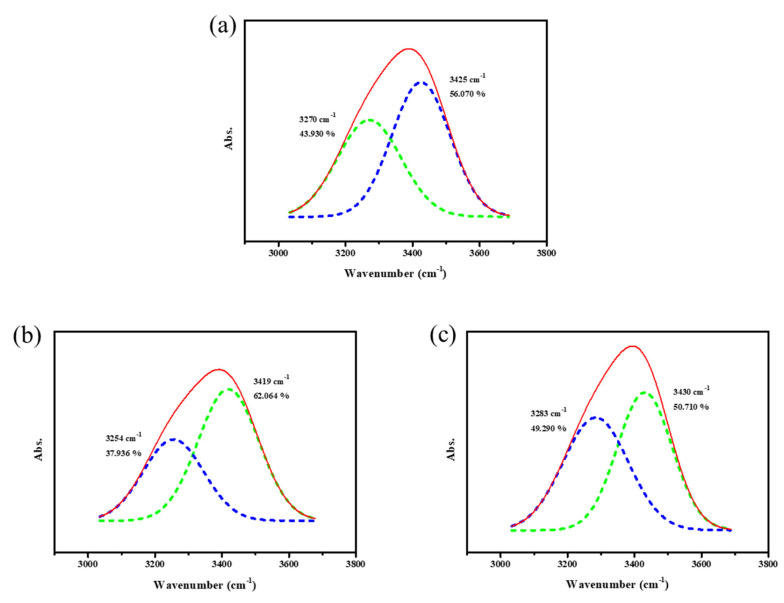
FT-IR: Deconvoluted FT-IR of hydroxy group of (**a**) neat HEC/PP, (**b**) HEC/LA/PP_A, and (**c**) HEC/LA/PP_B.

The hydroxy group was deconvolved to analyze the change in coordination in detail. (Figure 8 and Table 2) Comparing the PP/HEC and PP/HEC/LA_A, the peak of 3425 cm^−1^, the dominant peak of hydroxyl group, shifted to 3419 cm^−1^, and the area ratio increased from 56.070% to 62.064%. The peak of 3270 cm^−1^, a relatively low wavenumber, shifted to 3254 cm^−1^, and its area ratio decreased from 43.930% to 37.936% after the addition of the LA. This means that the LA was evenly dispersed and hindered the interactions between the HEC chains, resulting in weaker hydroxy bonding between the polymer chains. Thus, the vibrations of the OH bonds in each chain were enhanced. After the application of the vacuum-assisted method, the dominant peak shifted to 3430 cm^−1^ and the area ratio was partially restored, to 51%. The peak with the relatively low wavenumber shifted from 3254 cm^−1^ to 3283 cm^−1^, and the ratio of the area changed from 37.936% to 49.290%. It was confirmed that the rearrangement between the HEC polymer chains occurred, since the LA escaped. By comparing the wavenumbers of the neat HEC/PP and HEC/LA/PP_B, it was found that the overall increase was due to the escape of the LA, which caused the bond to be weaker than in the initial state, and the hydroxy bonding became stronger. The polymer chains tended to move into an enthalpy-stabilized state. As pores formed in the HEC region, rearrangement occurred, and the interaction between the chains was strengthened, maintaining the morphology of the HEC film.

#### 2.4.2. Ether Group

**Figure 9:**
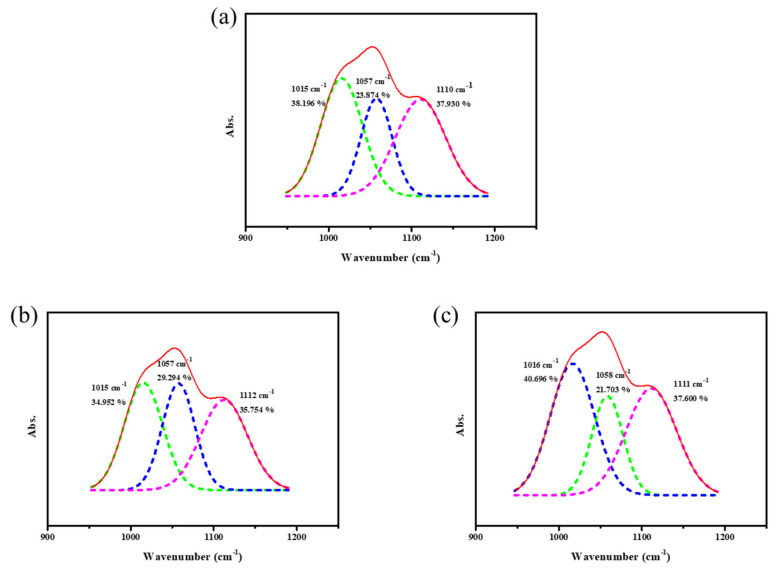
FT-IR: deconvoluted FT-IR of ether groups of (**a**) neat HEC/PP, (**b**) HEC/LA/PP_A, and (**c**) HEC/LA/PP_B.

Deconvolution was performed to analyze the change in the ether group in detail (Figure 9 and Table 3). Comparing the HEC/PP and HEC/LA/PP_A, there were few changes in the wavenumber overall. However, when the areas of each peak were compared, the area ratios of the peaks, with wavenumbers of 1057~1058 cm^−1^ and of 1110~1112 cm^−1^ increased, and the other peak decreased, resulting in a decrease in the difference between the area ratios. As the LA was added to the HEC polymer chains, the distance between the polymer chains increased and the interaction forces between the ether bonds in each chain weakened. This showed the same pattern as the change in the IR spectra of the hydroxy group of the HEC when the LA was added to the HEC. In addition, it was confirmed that after the process, the area ratio of the dominant peak increased from 38.196% to 40.696%, and the area ratio of the peaks with wavenumbers of 1057~1058 cm^−1^ decreased from 23.874% to 21.703%. However, even when the LA between the HEC chains escaped from the HEC/LA/PP membrane through the process, only slight changes in the wavenumbers of all the peaks were observed. This was considered to have been due to the fact that the ether group in the HEC could not affect the rearrangement of the HEC as the LA escaped from the HEC/LA/PP membrane during the process. Therefore, the rearrangement of the HEC seems to have occurred with the hydroxy group.

## 3. Experimental Methods

### 3.1. Materials

The 2-Hydroxyethyl cellulose (Mn: ~90,000 g/mol) was purchased from Sigma Aldrich CO (St. Louis, MO, USA). Acetone, glycerin, and lactic acid were purchased from Daejung Chemical and Metals (Siheung, Republic of Korea). Polypropylene prefilter which pore sizes of 0.6 μm was purchased from Merck Millipore Ltd. (Darmstadt, Germany) All chemicals were used as received.

### 3.2. Membrane Preparation

A 7-wt% HEC polymer solution was prepared by dissolving hydroxyethyl cellulose polymer into a co-solvent (Acetone: H_2_O = 8:2 wt/wt%) with 0.5 mol ratio of lactic acid per 1 mol of monomeric unit in HEC. This was stirred at room temperature using a stirrer for 18 h. The propylene support was soaked in 5 M of glycerin solution for 1 h and dried in a hot oven at 70 °C. The PP support was attached to the glass plate. The HEC/LA solution was coated on the polypropylene support to a thickness of 300 μm using a doctor blade. The residual solvent was removed through drying for 30 min at humidity conditions of both 25 °C and 50%.

### 3.3. Formation and Connection of Pores Using Vacuum-Assisted Process

**Scheme 1:**
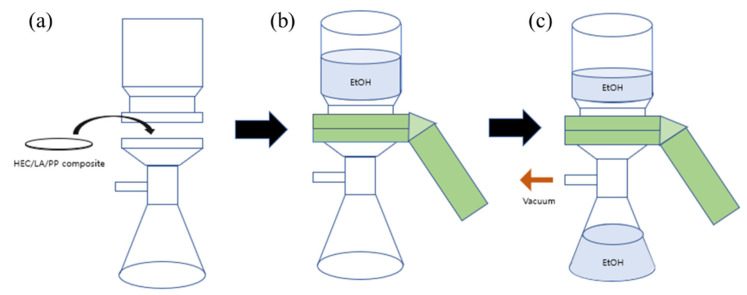
Vacuum-assisted process. The experiment was conducted by installing the experimental device and connecting it to the aspirator (**a**). The coating part was facing upward, so that the separator would not be damaged during the process (**b**). When a vacuum was applied, ethanol was forced to move vertically on the HEC side of the composite (**c**).

The prepared composite was carried out using to the vacuum-assisted process for pore formation such as Scheme 1. The experiment was conducted by installing the experimental device and connecting it to the aspirator (Scheme 1a). At this point, the coating part was facing upward, so that the separator would not be damaged during the process (Scheme 1b). Ethanol was used a mobile phase to form the pore in the composite instead of water since HEC is hydrophilic and the coating can be peeled off. When a vacuum was applied, ethanol was forced to move vertically on the HEC side of the composite (Scheme 1c). It pierced the plasticized part of the HEC and generated the formation of pores. The average ethanol-flux data of HEC/LA/PP composite were measured and expressed by LMH (L/m^2^h). The membrane, which was successfully tested, was dried in a vacuum oven for more than 4 days for analysis.

### 3.4. Characterization

To analyze the physicochemical properties of the membrane, TGA, FT-IR, and SEM were used. The surface of HEC/LA/PP membrane film was coated twice with magnetron sputter coater (MSC-101, JEOL, Seoul, Republic of Korea) and observed at a voltage of 16 kV with SEM (JSM-5600LV, JEOL, Seoul, Republic of Korea) magnified 400–5500 times. The TGA (Universal V4.5A, TA instrument, Seoul, Republic of Korea) was used to measure the thermal stability of the prepared HEC/LA/PP membrane, while the temperature was increased from room temperature to 800 °C at 10 °C per minute under a nitrogen atmosphere. The balance gas flow was 40.0 mL/min and the sample gas flow was 60.0 mL/min. The interaction force between the functional groups of HEC and LA was measured by scanning 16 times at a resolution of 4 cm^−1^ in the range of 4000 to 400 cm^−1^ wavenumber with a FT-IR spectrometer (VERTEX 70/70V FT-IR spectrometer, Bruker Optics, Gwangmyeng, Republic of Korea).

## 4. Conclusions

In this study, HEC-polymer-based experiments were conducted to improve the thermal stability and mechanical strength by using a PP as a support. In the SEM image, uniform nano-sized pores on the surface of the membrane were observed. Furthermore, it was confirmed that the HEC region of the membrane did not collapse before or after the coating process, even though pores formed, and it was observed that the pores in the PP region of the membrane were not blocked. To measure the thermal stability, an analysis was performed using a TGA instrument. The thermal stability was higher in the HEC/LA/PP membrane after the process than in the neat HEC and in the HEC/LA/PP composite before the process. The interaction between the HEC and the LA was analyzed using FT-IR. It was confirmed that before the process, the LA interfered with the interaction between the polymer chains, weakening the hydroxy bonding between them. After the process, the bond between the chains was restored through the rearrangement of the HEC, so that the film form could be maintained. Through the TGA and FT-IR analyses, it was confirmed that the HEC/LA/PP membrane adhered to the PP through the rearrangement of the HEC polymer. This increased the thermal stability of the composite.

As a result, we succeeded in making a porous membrane based on a HEC polymer with enhanced physicochemical properties by using a vacuum-assisted process. The success of the manufacturing of the vacuum-assisted-process-based membrane means that it is possible to manufacture battery separators more economically than with the hydraulic method over a large area, and that the pore size can be created uniformly. Furthermore, it is possible to manufacture a composite in which two polymers are attached without using additional adhesives. If this method were to be applied in the battery industry, additional processes and costs due to the introduction of adhesives would be reduced, and separators could be manufactured through a simple process.

## Figures and Tables

**Table 1:** Value of ethanol flux.

Category	Data
Average ethanol flux (L/m^2^h)	77.1
Standard deviation	5.3

**Table 2:** Area percentage of hydroxy group in FT-IR.

Wavenumber (cm^−1^)	HEC/PP	HEC/LA/PP_A.HEC	HEC/LA/PP_B.HEC
3254~3283 cm^−1^	43.930%	37.936%	49.290%
3419~3430 cm^−1^	56.070%	62.064%	50.710%

**Table 3:** Area percentage of ether group in FT-IR.

Wavenumber (cm^−1^)	HEC/PP	HEC/LA/PP_A.HEC	HEC/LA/PP_B.HEC
1015~1016 cm^−1^	38.196%	34.952%	40.696%
1057~1058 cm^−1^	23.874%	29.294%	21.703%
1110~1112 cm^−1^	37.930%	35.754%	37.600%

## Data Availability

Not applicable.

## Sample Availability

The samples are not available from the authors.

## References

[1] Bhute M.V., Kondawar S.B. (2019). Electrospun Poly(Vinylidene Fluoride)/Cellulose Acetate/AgTiO_2_ Nanofibers Polymer Electrolyte Membrane for Lithium Ion Battery. Solid State Ion..

[2] Liu J., Li W., Zuo X., Liu S., Li Z. (2013). Polyethylene-Supported Polyvinylidene Fluoride–cellulose Acetate Butyrate Blended Polymer Electrolyte for Lithium Ion Battery. J. Power Sources.

[3] Wei Z., Gu J., Zhang F., Pan Z., Zhao Y. (2020). Core–shell Structured Nanofibers for Lithium Ion Battery Separator with Wide Shutdown Temperature Window and Stable Electrochemical Performance. ACS Appl. Polym. Mater..

[4] Li A., Yuen A.C.Y., Wang W., De Cachinho Cordeiro I.M., Wang C., Chen T.B.Y., Zhang J., Chan Q.N., Yeoh G.H. (2021). A Review on Lithium-Ion Battery Separators Towards Enhanced Safety Performances and Modelling Approaches. Molecules.

[5] Goncalves R., Lizundia E., Silva M.M., Costa C.M., Lanceros-Méndez S. (2019). Mesoporous Cellulose Nanocrystal Membranes as Battery Separators for Environmentally Safer Lithium-Ion Batteries. ACS Appl. Energy Mater..

[6] Hu J., Liu Y., Zhang M., He J., Ni P. (2020). A Separator Based on Cross-Linked Nano-SiO_2_ and Cellulose Acetate for Lithium-Ion Batteries. Electrochim. Acta.

[7] Lv D., Chai J., Wang P., Zhu L., Liu C., Nie S., Li B., Cui G. (2021). Pure Cellulose Lithium-Ion Battery Separator with Tunable Pore Size and Improved Working Stability by Cellulose Nanofibrils. Carbohydr. Polym..

[8] Guo T., Song J., Jin Y., Sun Z., Li L. (2019). Thermally Stable and Green Cellulose-Based Composites Strengthened by Styrene-Co-Acrylate Latex for Lithium-Ion Battery Separators. Carbohydr. Polym..

[9] Sun X., Li M., Ren S., Lei T., Lee S.Y., Lee S., Wu Q. (2020). Zeolitic Imidazolate Framework-Cellulose Nanofiber Hybrid Membrane as Li-Ion Battery Separator: Basic Membrane Property and Battery Performance. J. Power Sources.

[10] Yang Y., Huang C., Gao G., Hu C., Luo L., Xu J. (2020). Aramid Nanofiber/Bacterial Cellulose Composite Separators for Lithium-Ion Batteries. Carbohydr. Polym..

[11] Zhang L., Li X., Yang M., Chen W. (2021). High-Safety Separators for Lithium-Ion Batteries and Sodium-Ion Batteries: Advances and Perspective. Energy Storage Mater..

[12] Jeon H., Yeon D., Lee T., Park J., Ryou M.H., Lee Y.M. (2016). A Water-Based Al_2_O_3_ Ceramic Coating for Polyethylene-Based Microporous Separators for Lithium-Ion Batteries. J. Power Sources.

[13] Wu D., Dong N., Wang R., Qi S., Liu B., Wu D. (2021). In Situ Construction of High-Safety and Non-Flammable Polyimide “Ceramic” Lithium-Ion Battery Separator via SiO_2_ Nano-Encapsulation. Chem. Eng. J..

[14] Xie W., Liu W., Dang Y., Tang A., Deng T., Qiu W. (2019). Investigation on Electrolyte-Immersed Properties of Lithium-Ion Battery Cellulose Separator through Multi-Scale Method. J. Power Sources.

[15] Zhang H., Ren Q., Mohd S., Yang C., Li J., Pei Y., Luo X. (2021). Early-Warning and Semi-Quantitative Colorimetric Detection of Hg (II) with Lysine-Bis-Schiff Base Cellulose Membranes Designed by Simple Interfacial Covalent Bonding. Sens. Actuators B Chem..

[16] Wu J., Yuan Q. (2002). Gas Permeability of a Novel Cellulose Membrane. J. Membr. Sci..

[17] Choudhury P.R., Majumdar S., Sahoo G.C., Saha S., Mondal P. (2018). High Pressure Ultrafiltration CuO/Hydroxyethyl Cellulose Composite Ceramic Membrane for Separation of Cr (VI) and Pb (II) from Contaminated Water. Chem. Eng. J..

[18] Jung J.T., Kim J.F., Wang H.H., Di Nicolo E., Drioli E., Lee Y.M. (2016). Understanding the Non-Solvent Induced Phase Separation (NIPS) Effect during the Fabrication of Microporous PVDF Membranes via Thermally Induced Phase Separation (TIPS). J. Membr. Sci..

[19] Apel P. (2001). Track Etching Technique in Membrane Technology. Radiat. Meas..

[20] Rhyu S.Y., Kang S.W. (2021). Accelerated CO_2_ Transport by Synergy Effect of Ionic Liquid and Zn Particles. J. Ind. Eng. Chem..

[21] Lee W.G., Cho Y., Kang S.W. (2020). Effect of Ionic Radius in Metal Nitrate on Pore Generation of Cellulose Acetate in Polymer Nanocomposite. Polymers.

[22] Ma X., Zuo X., Wu J., Deng X., Xiao X., Liu J., Nan J. (2018). Polyethylene-Supported Ultra-Thin Polyvinylidene Fluoride/Hydroxyethyl Cellulose Blended Polymer Electrolyte for 5 V High Voltage Lithium Ion Batteries. J. Mater. Chem..

[23] Wijayasinghe R., Vasiljevic T., Chandrapala J. (2015). Water-Lactose Behavior as a Function of Concentration and Presence of Lactic Acid in Lactose Model Systems. J. Dairy Sci..

[24] Kim S.H., Kang S.W. (2021). Preparation of Highly Stable Cellulose Separator by Incorporation of Lactic Acid. Cellulose.

[25] Li L., Fan E., Guan Y., Zhang X., Xue Q., Wei L., Wu F., Chen R. (2017). Sustainable Recovery of Cathode Materials from Spent Lithium-Ion Batteries using Lactic Acid Leaching System. ACS Sustain. Chem. Eng..

